# Irrigant flow in the root canal during ultrasonic activation: A numerical fluid–structure interaction model and its validation

**DOI:** 10.1111/iej.13791

**Published:** 2022-07-14

**Authors:** Christos Boutsioukis, Bram Verhaagen, Lucas W. M. van der Sluis, Michel Versluis

**Affiliations:** ^1^ Department of Endodontology, Academic Centre for Dentistry Amsterdam (ACTA) University of Amsterdam and Vrije Universiteit Amsterdam Amsterdam The Netherlands; ^2^ Physics of Fluids Group, Faculty of Science and Technology University of Twente Enschede The Netherlands; ^3^ Center for Dentistry and Oral Hygiene, University Medical Center Groningen University of Groningen Groningen The Netherlands; ^4^ Technical Medical (TechMed) Centre and MESA^+^ Institute for Nanotechnology University of Twente Enschede The Netherlands

**Keywords:** Computational Fluid Dynamics, high‐speed imaging, irrigation, Particle Image Velocimetry, ultrasonic activation

## Abstract

**Aim:**

The aim of the study was (a) to develop a three‐dimensional numerical model combining the oscillation of a tapered ultrasonic file and the induced irrigant flow along with their two‐way interaction in the confinement of a root canal. (b) To validate this model through comparison with experiments and theoretical (analytical) solutions of the flow.

**Methodology:**

Two partial numerical models, one for the oscillation of the ultrasonic file and another one for the irrigant flow inside the root canal around the file, were created and coupled in order to take into account the two‐way coupled fluid–structure interaction. Simulations were carried out for ultrasonic K‐files and for smooth wires driven at four different amplitudes in air or inside an irrigant‐filled straight root canal. The oscillation pattern of the K‐files was determined experimentally by Scanning Laser Vibrometry, and the flow pattern inside an artificial root canal was analysed using high‐speed imaging together with Particle Image Velocimetry. Analytical solutions were obtained from an earlier study. Numerical, experimental and analytical results were compared to assess the validity of the model.

**Results:**

The comparison of the oscillation amplitude and node location of the ultrasonic files and of the irrigant flow field showed a close agreement between the simulations, experiments and theoretical solutions.

**Conclusions:**

The model is able to predict reliably the file oscillation and irrigant flow inside root canals during ultrasonic activation under similar conditions.

## INTRODUCTION

Ultrasonic irrigant activation is probably the most widely used supplementary irrigation method (Dutner et al., 2012; van der Sluis et al., 2007; Willershausen et al., 2015). It aims to improve debridement and disinfection in difficult‐to‐reach areas of the root canal system, such as isthmuses, uninstrumented oval extensions, fins and accessory canals (Haapasalo et al., 2010). Its cleaning effect has been attributed to the file‐induced microstreaming and possibly also to cavitation produced by an instrument oscillating at ultrasonic frequency inside an irrigant‐filled root canal (Ahmad et al., 1987a, 1987b; Macedo et al., 2014; Robinson et al., 2018; Verhaagen et al., 2014). *Ex vivo* and *in vitro* studies have shown that ultrasonic activation is able to remove pulp tissue remnants and dentin debris from root canals more effectively than syringe irrigation but conflicting findings have been reported regarding its antimicrobial effect (Căpută et al., 2019). In addition, widely varying activation protocols have been employed without a clear consensus on the most effective approach (Căpută et al., 2019). A detailed investigation of the underlying physical mechanisms and especially of the developed irrigant flow during ultrasonic activation could provide further insight and lead to an optimized irrigation technique.

Previous studies have assessed the oscillation characteristics of ultrasonic files (Ahmad et al., 1993; Lea et al., 2010; Verhaagen et al., 2012) and have also investigated the irrigant flow during ultrasonic activation in experiment (Jiang et al., 2010a; Malki et al., 2012; Roy et al., 1994; Verhaagen et al., 2014). Despite the valuable information obtained, experiments can only provide a partial understanding of the flow and the related phenomena inside root canals due to the associated small time and length scales and the three‐dimensional nature of the flow. In addition, certain quantities such as the pressure and shear stress on the root canal wall are difficult to measure using current measurement techniques.

Numerical models have been used in the past to obtain relevant information about the irrigant flow inside root canals during syringe irrigation (Boutsioukis et al., 2009, 2010; Shen et al., 2010; Wang et al., 2015). A simplified two‐dimensional model of the file‐induced microstreaming inside a root canal during ultrasonic activation employing one‐way fluid–structure coupling (the oscillation was prescribed explicitly instead of being calculated by the model) has also been developed and validated as a first step (Verhaagen et al., 2014). However, due to the complex flow having significant components in the apico‐coronal direction, it was concluded that a full three‐dimensional approach is required to resolve the complete picture. An earlier study used a three‐dimensional model, but the oscillation of the ultrasonic tip was also prescribed explicitly and the damping effect of the surrounding irrigant on the file oscillation was not taken into account (Chen et al., 2014). In addition, the model was not validated, a procedure that is considered essential for all numerical models (Oberkampf & Trucano, 2002).

Therefore, the aim of this study was twofold: (a) to develop a three‐dimensional numerical model combining the computed oscillations of an ultrasonic file and the induced fluid flow along with their two‐way interaction in the confinement of a simplified root canal, (b) to validate this model through a comparison with experiments and theoretical (analytical) solutions of the same flow (Verhaagen et al., 2014).

## MATERIALS AND METHODS

### Computational models

A partitioned approach was used in order to simulate the flow around an oscillating ultrasonic file. Two partial models, one for the oscillation of the ultrasonic file (*structural model*) and another one for the irrigant flow inside the root canal around the file (*flow model*), were created within the Workbench 14.5 environment (ANSYS Inc.) and were coupled in order to take into account the two‐way coupled fluid–structure interaction (FSI) between the file and the surrounding irrigant.

#### Structural model

The geometry of size 15/.02 taper and 25‐mm ultrasonic K‐files (Acteon Satelec) was obtained through examination under a stereoscopic microscope (Stemi SV‐6, Zeiss) at 10× to 40× magnification. In addition, the files were sectioned at various levels perpendicular to their long axis, embedded in self‐curing acrylic resin (Vertex Self‐curing, Vertex Dental), polished and examined under a scanning electron microscope (XL‐20, Philips) at 250× magnification to obtain the cross‐sectional shape. All photos were analysed in Fiji 1.46 m (Schindelin et al., 2012) to determine the three‐dimensional shape of the files. Their length was determined with precision callipers (accuracy 0.05 mm).

**FIGURE 1:**
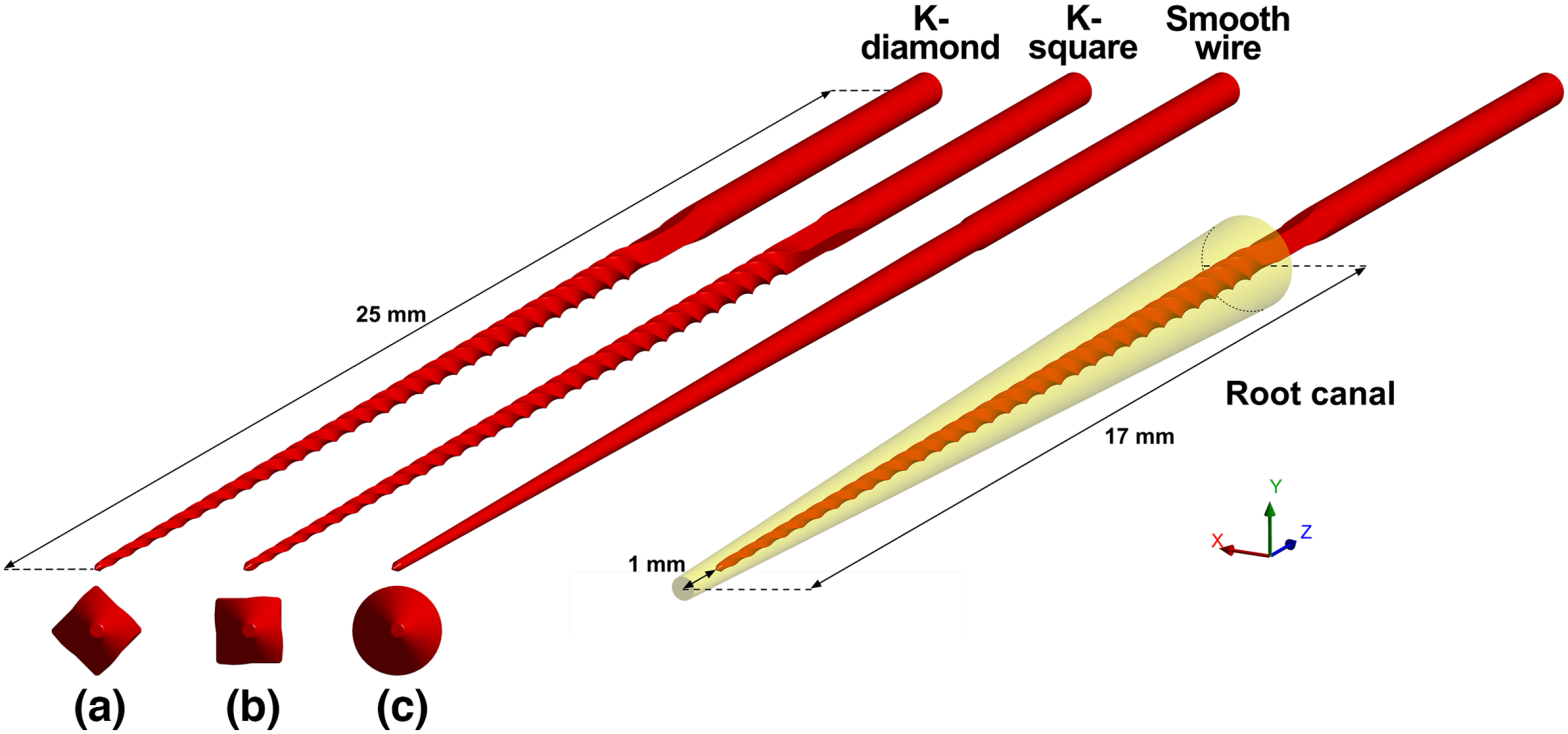
Geometrical characteristics of the structural and flow models used. The files/wires were driven to oscillate in the *y*‐direction.

The preprocessor Design Modeller 14.5 (ANSYS Inc.) was used to recreate the three‐dimensional geometry of the K‐file (Figure 1) and Mesh 14.5 (ANSYS Inc.) was used to create a hybrid mesh of approximately 88 000 elements and 60 000 nodes, which included mainly hexahedral and tetrahedral elements and a small number of pyramidal elements (0.2%). Grid‐independence of the results was verified. The exact orientation of the file cross‐section in relation to the primary oscillation plane (Jiang et al., 2010a) varied in different file specimens examined under the microscope, so two different cases were studied in this model; the file oscillated along one of the cross‐section diagonals (K‐diamond case; Figure 1a) or it was rotated 45° around its long axis to oscillate parallel to two of the sides of its square cross‐section at the tip (K‐square case; Figure 1b). One additional type of instrument was studied, namely a tapered smooth wire having the same size, taper and length as the K‐file but with a circular cross‐section (Figure 1c). This instrument was included to reduce the complexity of the numerical simulation so as to facilitate comparison of the simulation results to theoretical solutions; smooth ultrasonic tips of similar dimensions are also used clinically for irrigant activation.

All files/wires were assumed to be made of homogeneous and isotropic stainless steel AISI type 304 with a density of 7900 kg/m^3^, Young's modulus of 195 GPa and Poisson's ratio of 0.29 (Aerospace Specification Metals, 2012; Lide, 2005). Each file/wire was driven laterally along the *y*‐direction (perpendicular to its long axis) at its distal end (shank) by a periodic sinusoidal displacement *y* = *y*_o_·sin(2*π*·*f*·*t*) at frequency *f* = 30.5 kHz and using different driving amplitudes (*y*_o_ = 2.5, 5.0, 7.5, or 10.0 μm). These parameters corresponded to experimentally obtained values (Verhaagen et al., 2012). Constraints were added to prevent any movement of the distal end in the other directions. The rest of the file was allowed to oscillate freely and develop its oscillation pattern as a result of the driving and the interaction with the surrounding irrigant, when present, that is there was no prescribed motion. The surface of the file/wire that was immersed in the irrigant within the root canal was defined as the FSI boundary where displacements and forces would be exchanged between the two models. As initial conditions, the file was assumed to be at rest with zero velocity and displacement, and thus such transients of the file motion were included in the simulations.

The Transient Structural 14.5 module (ANSYS Inc.), a finite element solver, was used for the structural calculations. Non‐linear dynamic equilibrium equations were solved by implicit integration schemes. Force and displacement were monitored to ensure convergence. A time step of 3.28 × 10^−7^ s was used throughout the numerics, which equals 1/100^th^ of the oscillation period (*T* = 3.28 × 10^−5^ s).

#### Flow model

A simplified straight root canal was simulated as a geometrical frustum of a cone. No apical constriction or irregularities were included in the model because they were beyond the scope of this study. The length of the root canal was 17 mm and the diameter was 0.35 mm (ISO size 35) at the apical end and 1.37 mm at the canal orifice (6% taper; Figure 1). The file/wire was inserted at 1 mm from the apical end of the root canal and it was centred. The preprocessors Design Modeller 14.5 (ANSYS Inc.) and Mesh 14.5 (ANSYS Inc.) were used to create the three‐dimensional geometry of the root canal and the hybrid mesh of the flow domain (approximately 1.4 million cells, predominantly tetrahedral and a small number of prismatic cells). The mesh was refined near the file/wire surface (FSI boundary) and near the root canal wall. Grid‐independence of the results was verified.

The apical terminus of the root canal and the lateral wall were simulated as rigid, smooth and impermeable walls; a no‐slip boundary condition was imposed on these walls. A pressure‐outlet boundary condition was applied to the coronal orifice (atmospheric pressure). The root canal was assumed to be filled with 1% NaOCl solution, which was modelled as an incompressible Newtonian fluid with a density of 1040 kg/m^3^ and viscosity 0.986 × 10^−3^ Pa s (Guerisoli et al., 1998). No chemical activity was included in the present simulations. The irrigant was initially at rest. Gravity was included in the flow field in the direction of the positive z axis.

The Fluent 14.5 module (ANSYS Inc.), which is a finite volume solver, was used to solve the time‐dependent Navier–Stokes equations. The Dynamic Mesh capability of the solver, which is based on the Arbitrary Lagrange–Euler formulation, was employed to account for the motion of the FSI boundary (surface of the ultrasonic file). The fluid mesh was updated at the beginning of every time step through local remeshing and smoothing in order to follow the moving boundary. An unsteady isothermal flow was assumed and no turbulence model was used because the flow was outside the bounds of turbulence. All transport equations were discretized to be at least second‐order accurate in space, while a first order implicit formulation was used for temporal discretization. The convergence criterion was set to 10^−4^ of the maximum scaled residuals. Pressure and velocity in selected areas of the flow domain were also monitored to ensure adequate convergence at every time step. A time step of 3.28 × 10^−7^ s was used, similar to the structural model.

#### Coupling

The two‐way serial coupling between the fluid and structure models was realized through the coupling module (ANSYS Inc.). Each time step was divided in a number of sub‐steps (coupling iteration loop). The two solvers exchanged data about the node positions and forces on the FSI boundary at the beginning of each sub‐step, then calculated the solutions for the solid and fluid domain separately and then exchanged data again. If convergence was reached the solvers proceeded to the next time step, otherwise a new sub‐step iteration was initiated. This process was continued until the solutions converged for all time steps.

In order to ensure reasonable use of computation resources, two additional cases involving the K‐file (K‐diamond orientation) driven at the maximum amplitude (10 μm) were used to determine the optimum end time of the simulations. The first case included the complete K‐file and root canal and the calculations were continued for 30 oscillation cycles. The second case included the complete K‐file but only the apical 5 mm of the root canal, in order to study the evolution of the flow in the apical third for a longer period of time. The calculations were continued for 100 oscillation cycles in this case. The time‐averaged velocity in the apical third of the root canal was compared at various time points. Based on the results, the main simulations were continued for 20 cycles (*t* = 0.656 ms), since at that time point a quasi‐steady state was reached. Computations were carried out on a Z620 workstation (Hewlett‐Packard) with a 6‐core Intel Xeon 3.2 GHz processor (Intel) and 32 GB of RAM.

#### Cases studied and data analysis

Each of the three different files/wires (K‐diamond, K‐square, smooth wire) were driven at four different amplitudes (2.5, 5.0, 7.5 and 10.0 μm) assuming either free oscillation without the presence of the root canal and irrigant or oscillation inside an irrigant‐filled root canal. Twenty‐four cases were studied in total (not including the two cases that were studied to determine the optimum end time). The oscillation pattern of the file/wire was extracted and analysed in Excel 2010 (Microsoft). The irrigant velocity field and the pressure on the root canal wall were also exported; time‐averaging was conducted in MATLAB R2015b (The MathWorks).

### Experiments

#### High‐speed imaging and Particle Image Velocimetry

An artificial transparent root canal was fabricated using polydimethylsiloxane (PDMS; Sylgard 184, Silicone Elastomer kit; Dow Corning) and a modified D‐size finger spreader (Dentsply Maillefer) with a tip diameter of 0.35 mm (ISO size 35) and a taper of 6%. The artificial root canal had a total length of 17 mm. These dimensions matched the root canal simulated in the numerical model.

A size 15/.02 taper and 25‐mm ultrasonic K file (Acteon Satelec) was attached to an ultrasonic handpiece (P‐Max Newtron, Acteon Satelec), which was mounted on a micrometric translation stage (9067 M, New Focus and M‐044.00, Physik Instrumente) capable of adjusting its position in three directions and tilting around two axes. Using this setup the file was positioned stably inside the root canal at 1 mm from the apex and centred along its main axis so that it could not touch the root canal wall during oscillation. The root canal was filled with distilled water (irrigant). Activation was performed with an ultrasound device (P‐Max Newtron, Acteon Satelec) operated at a power setting of ‘Green 5’ and ‘Yellow 5’ (approximately 22.5% and 46.3% of maximum power, respectively). Monodisperse hollow glass spheres of 10 μm diameter (Sphericel, Potters Industries; mean density of 1.1 × 10^3^ kg/m^3^, Stokes number in the order of one for the highest velocities expected) were added to the irrigant for flow quantification purposes.

The streaming around the ultrasonic file was recorded using a high‐speed camera (HPV‐1, Shimadzu Corp.), capable of recording 100 frames at speeds up to 10^6^ frames per second (Versluis, 2013). The camera was attached to a microscope (BX‐FM, Olympus Corp.) with a 20× magnification objective lens. The measurement depth of this setup was approximately 70 μm. The objective was focused on the centre longitudinal plane of the file and root canal guided by the major perimeter of the file at rest. Illumination for bright field imaging was provided by a high‐intensity light source (ILP1, Olympus Corp.). The file was driven to oscillate in the imaging plane in order to obtain side view velocity fields and it was allowed to oscillate for at least 1 s before any measurement took place to skip the transient start‐up phase. The high‐speed recordings were analysed using a Particle Image Velocimetry (PIV) algorithm with ensemble‐averaging (MATLAB, The Mathworks) that was developed in‐house. The irrigant flow field was extracted from these recordings.

#### Scanning Laser Vibrometry

The oscillation characteristics of a size 15/.02 taper and 25‐mm ultrasonic K‐file (Acteon Satelec) in air and inside a size 35/.06 taper PDMS root canal filled with tap water were recorded by a scanning laser vibrometer (OFV056, Polytec) at least 1 s after the beginning of the oscillation to skip the transient start‐up phase. The file was placed at 3 mm from the apical end, and it was driven by an ultrasound device (P‐Max Newtron, Acteon Satelec) operated at a power setting of ‘Green 5’, ‘Yellow 5’ and ‘Blue 5’ (approximately 22.5%, 46.3% and 75% of maximum power, respectively). These experiments have been described in detail in an earlier study (Verhaagen et al., 2012). Data on the oscillation amplitude along the file as a function of the driving amplitude were exported and analysed in Excel 2010 (Microsoft).

#### Theoretical (analytical) solution

The theoretical description of microstreaming around an ultrasonic file (analytical solution) has also been detailed in a previous study (Verhaagen et al., 2014). Briefly, the velocity (*u*_*s*_) of the steady irrigant jet created by the interaction of the inner and outer boundary layer around an oscillating smooth wire with a circular cross‐section scales quadratically with the tip oscillation amplitude (*A*) according to the equation:

(1) $$u_{s}=-\frac{3}{4}\frac{\omega A^{2}}{R}$$

where *ω* = 2π·*f* is the angular frequency of the oscillation and *R* is the radius of the wire cross‐section.

## RESULTS

### Validation of the structural model

**FIGURE 2:**
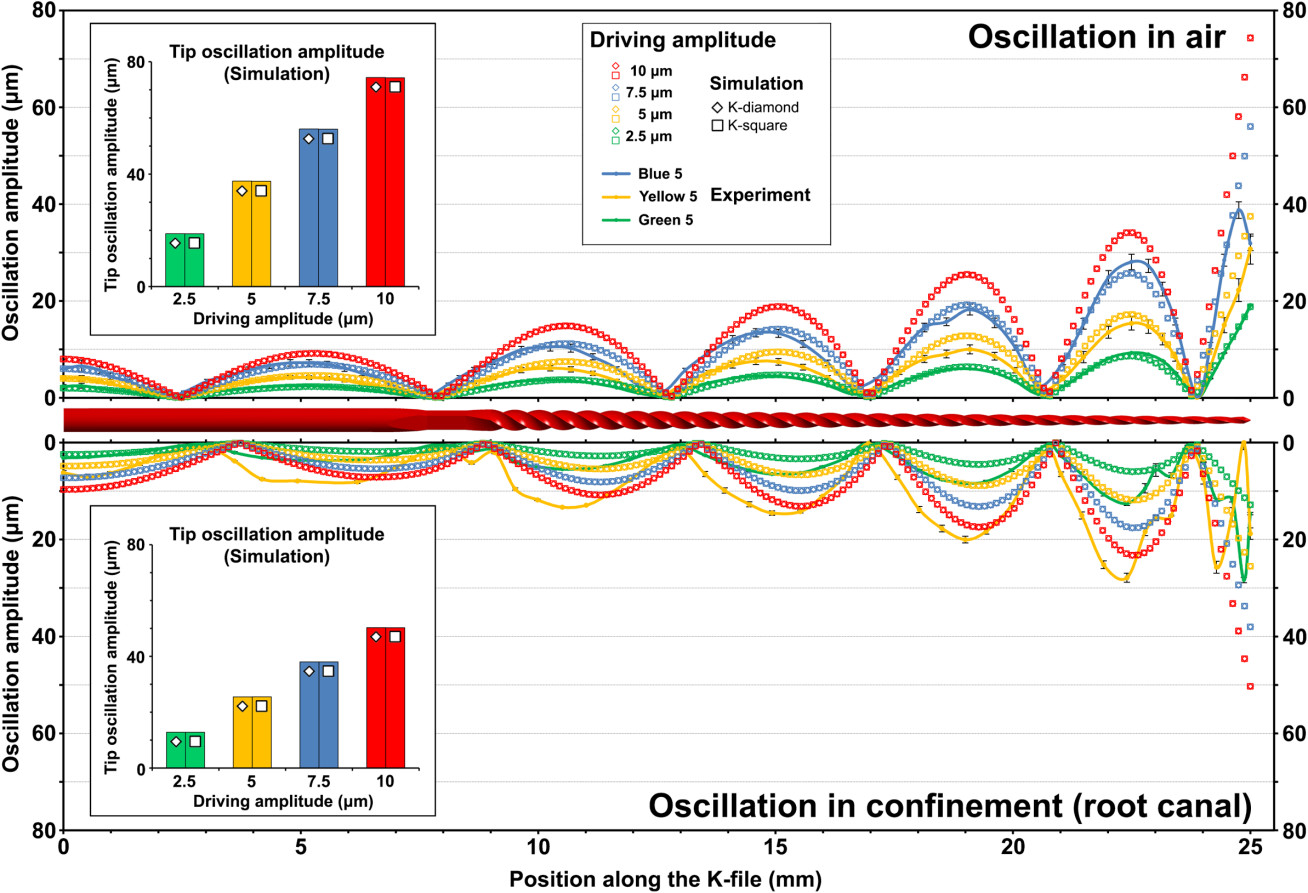
*Upper half*: Oscillation pattern of the K‐files at the time of maximum tip amplitude according to the numerical simulations in the absence of the root canal and irrigant, and compared with scanning laser vibrometer measurements on K‐files oscillating in air. Error bars indicate standard deviation. The two K‐file tip orientations (diamond and square) gave almost identical results. The ‘Green‐5’ and ‘Yellow‐5’ power settings of the ultrasound device corresponded to 2.5 and 5 μm driving amplitude in the simulations. *Lower half*: Oscillation pattern of the K‐files including the effect of the surrounding root canal and irrigant, compared to vibrometer measurements on K‐files oscillating inside irrigant‐filled root canals. The ‘Green‐5’ and ‘Yellow‐5’ power settings of the ultrasound device corresponded to 5 and 10 μm driving amplitude in the simulations. *Inserts*: Oscillation amplitude at the tip of the K‐files as a function of driving amplitude according to the simulations. The presence of the root canal and irrigant caused a notable decrease in the amplitude.

Comparison of the oscillation pattern of the K‐files as calculated by the structural model alone (without the root canal and the irrigant) with the oscillation pattern of K‐files oscillating in air as recorded by scanning laser vibrometry showed that there is a good match in terms of node position and oscillation amplitude at the antinodes, with the exception of the file tip where differences up to 20 μm were noted (Figure 2). The cases using 2.5, 5.0 and 7.5 μm driving amplitude in the simulations corresponded well to the cases using ‘Green 5’, ‘Yellow 5’ and ‘Blue 5’ power setting in the experiments. An increase in the driving amplitude (simulations) or power setting (experiments) led to an increase in the oscillation amplitude, while the position of the nodes and antinodes was generally unaffected. Oscillations were verified to have achieved a quasi‐stable situation already after two cycles in the simulation.

When the interaction with the root canal and irrigant was taken into account, the position of the nodes and the oscillation amplitude at the antinodes changed compared with the case in air. The oscillation amplitude of the K‐files, as calculated by the numerical model, was 32% smaller than the freely oscillating files due to viscous damping by the surrounding liquid. However, the oscillation amplitude measured during the experiments inside the root canal was larger compared with the same cases examined without confinement even though identical power settings were used. The cases with 5 and 10 μm driving amplitude in the simulations corresponded to the cases using ‘Green 5’ and ‘Yellow 5’ driving amplitude in the experiments (Figure 2). Small differences between simulations and experiments were again noted near the file tip. The two K‐file orientations simulated by the model (K‐diamond and K‐square) gave almost identical results in all cases regarding the oscillation pattern, with differences ranging from 0.02% to 0.13% in the tip oscillation amplitude (Figure 2). The oscillation amplitude of the smooth wire in the simulations was largely unaffected by the confinement of the root canal and irrigant.

### Validation of the flow model

**FIGURE 3:**
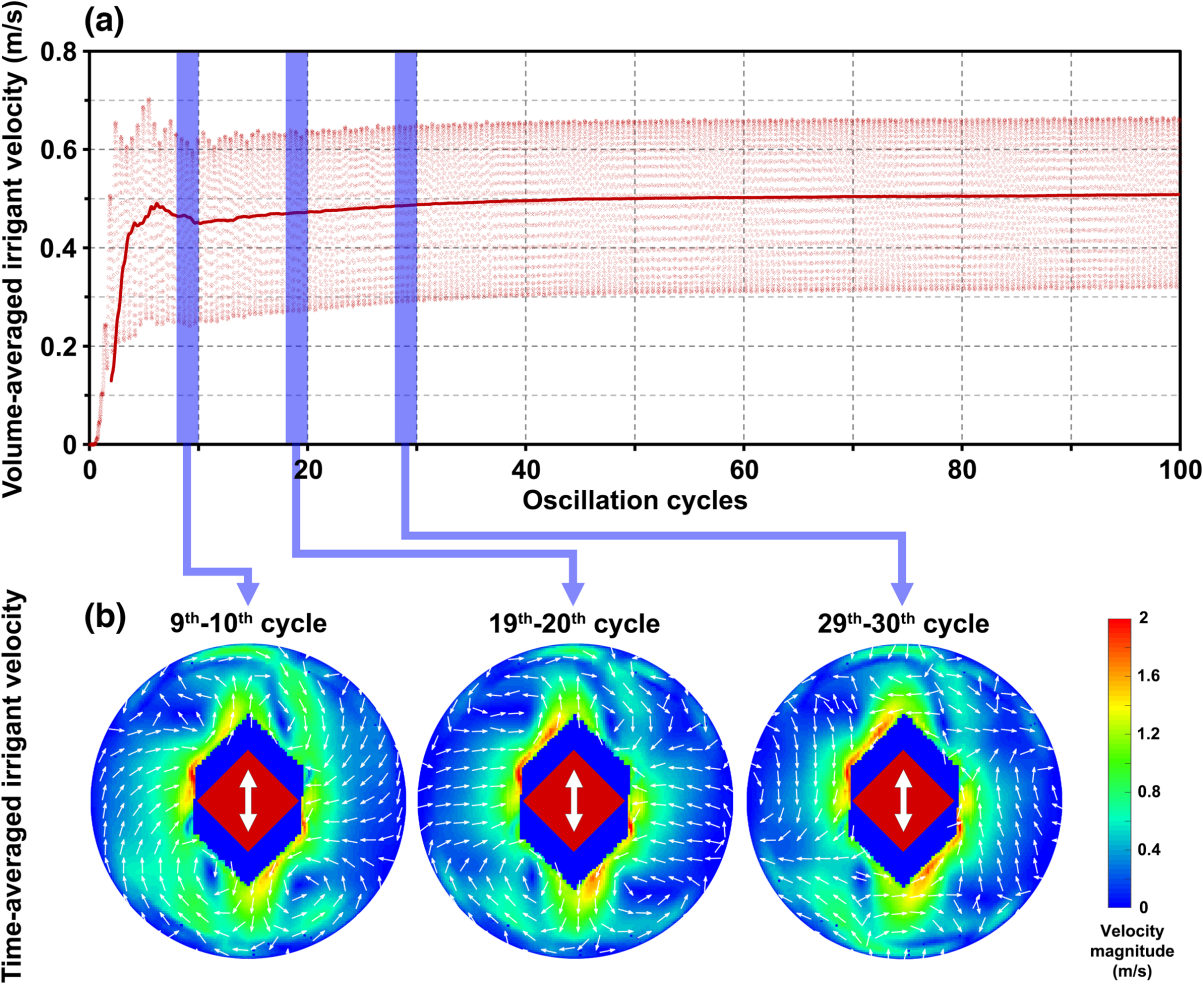
(a) Volume‐averaged irrigant velocity in the apical third of the root canal during the first 100 cycles according to numerical simulations by the partial root canal model (apical 5 mm of the root canal). Each dot represents one time step (1/100th of a cycle). The solid line depicts a moving average over the previous 2 cycles. The average velocity appears to reach a steady state after 20–30 cycles with little change after that. (b) Time‐averaged irrigant velocity at a cross‐section of the root canal at the beginning of the twisted part of the K‐file (150 μm from its tip) according to numerical simulations using the complete root canal model (driving amplitude = 10 μm). The direction of main oscillation is depicted by the white arrows. The dark coloured area in the middle of each plot is the area occupied by the K‐file (in red) during oscillation. No time‐averaging took place in that area. A comparison revealed only minor differences between the 19th–20th and 29th–30th cycle flow fields.

The volume‐averaged velocity of the irrigant in the apical third of the root canal (Figure 3a) and the time‐averaged flow fields (Figure 3b) showed that the flow continued to develop after the first 10 cycles. The differences between the time‐averaged flow fields in the 19th–20th and 29th–30th cycles were sufficiently small to consider the end of the 20th cycle (*t* = 6.56 × 10^−4^ s) to be the optimum end time of the simulations (steady‐state regime). Therefore, all time‐averaged quantities were calculated by averaging over the 19th and 20th cycle.

**FIGURE 4:**
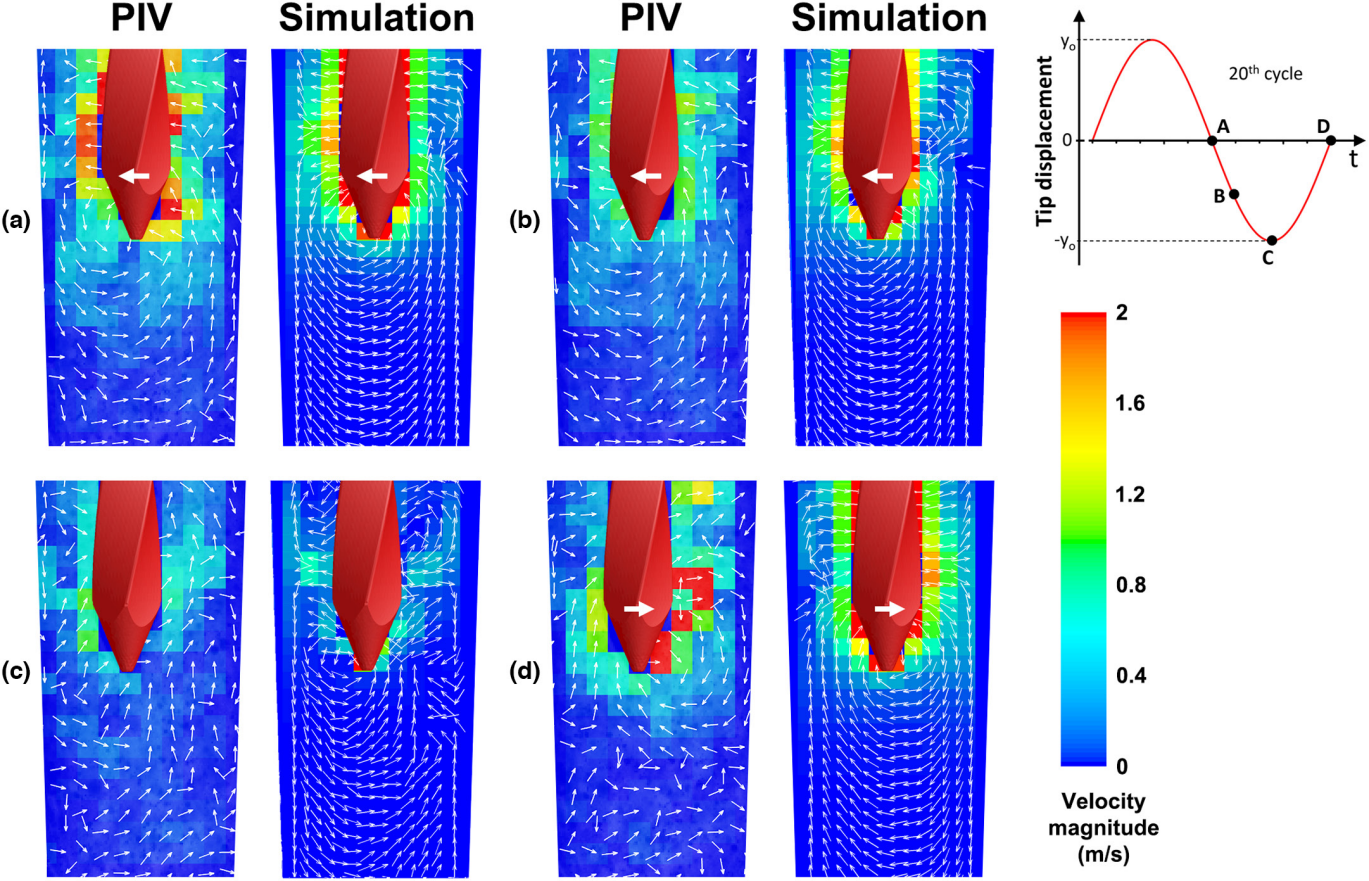
Instantaneous irrigant velocity contours and vectors along the main oscillation plane (*y*–*z* plane) near the tip of the K‐file, at time points a, b, c and d (see time‐reference graph on the right), according to PIV experiments (power setting ‘yellow 5’) and numerical simulations (driving amplitude = 5 μm). The direction of file motion is depicted by the white arrows on the K‐files.

**FIGURE 5:**
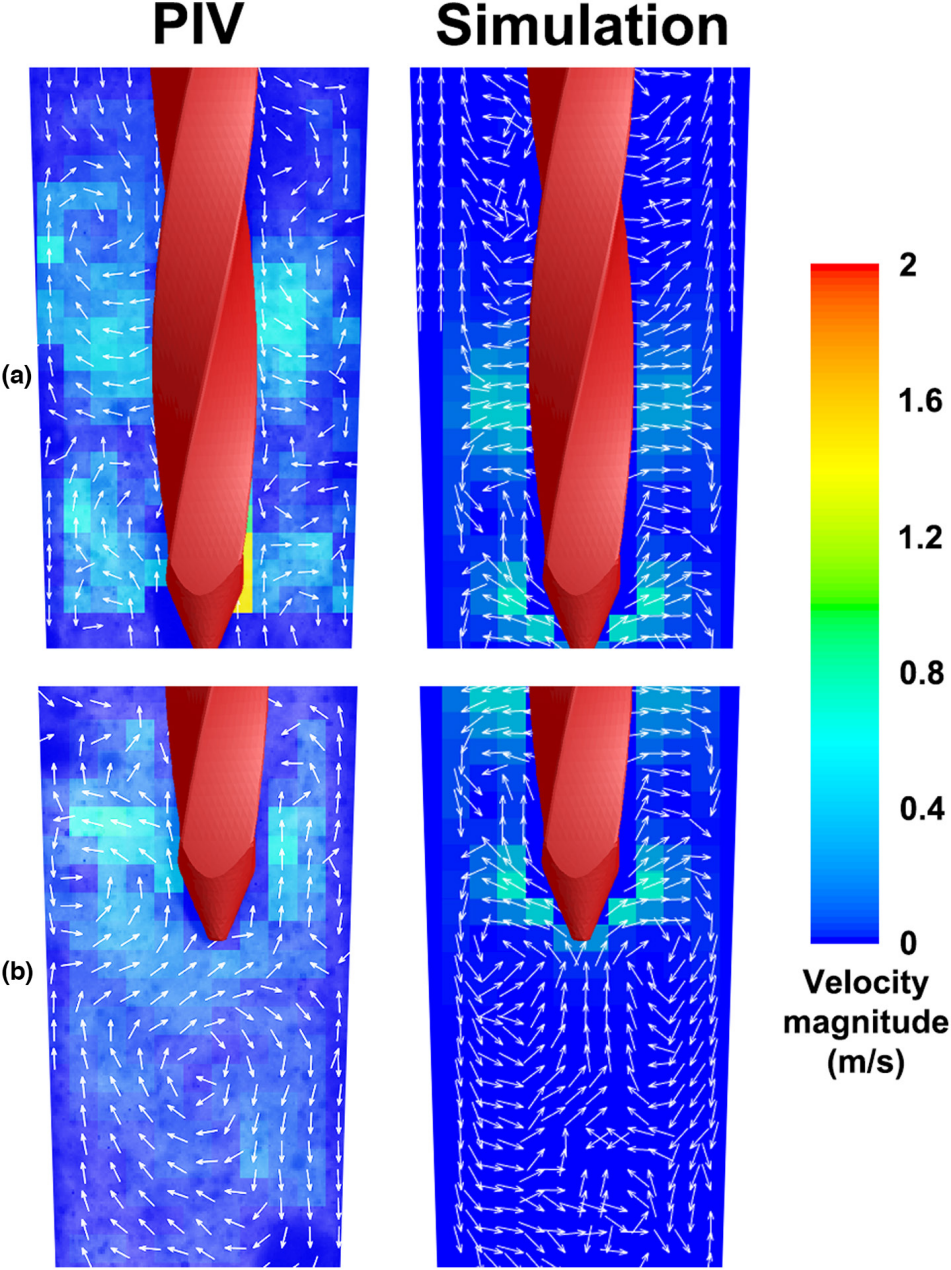
Time‐averaged irrigant velocity contours and vectors near the tip of the K‐file along the main oscillation plane (*y*–*z* plane), according to PIV experiments (power setting ‘green 5’) and numerical simulations (driving amplitude = 5 μm). The numerical data are depicted following down‐sampling to match the resolution of the PIV data. The comparison between the experiments and simulations revealed many phenomenological similarities regarding the flow pattern and the location and value of the maximum velocity.

The simulated instantaneous flow field around the K‐file tip in side view (along the main oscillation plane *y*–*z*) during the 20th oscillation cycle (Movie S1) corresponded to the PIV measurements in terms of velocity magnitude and direction (Figure 4). Comparison of the simulated time‐averaged flow field with the time‐averaged PIV‐determined flow field also revealed many phenomenological similarities regarding the flow pattern and the location and value of the maximum velocity (Figure 5).

**FIGURE 6:**
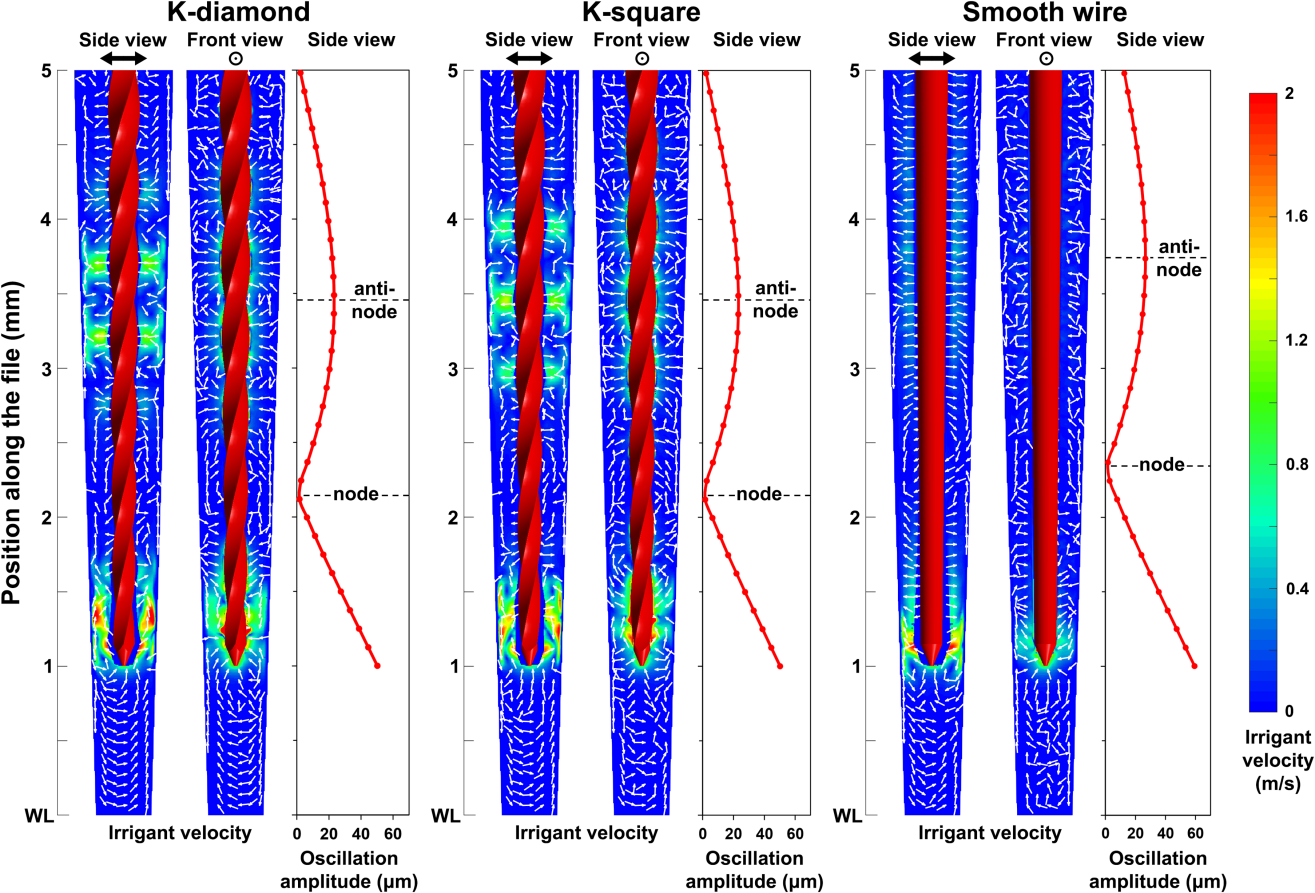
Time‐averaged irrigant velocity along the main oscillation plane (*y*–*z* plane; side view) and in the direction perpendicular to the main oscillation plane (*x*–*z* plane; front view) in the apical part of the root canal and modulus of the oscillation amplitude of the ultrasonic file according to numerical simulations (driving amplitude = 10 μm). The orientation of the ultrasonic file is given for comparison. Note that the areas of high irrigant velocity in the side view correspond to the parts of the K‐file where the twisted sharp edges were aligned with the main oscillation plane near the second antinode. This was less pronounced in the case of the smooth wire, which has no sharp edges.

With respect to the simulated flow pattern, areas of high time‐averaged irrigant velocity around the K‐file corresponded closely to the antinodes of the K‐file, with maximum values appearing in areas where the sharp edges of the K‐files were aligned with the main oscillation direction (*y*–*z* plane) near an antinode (Figure 6). A similar but less pronounced pattern was observed in the case of the smooth wire. This match confirmed the validity of the coupling process.

**FIGURE 7:**
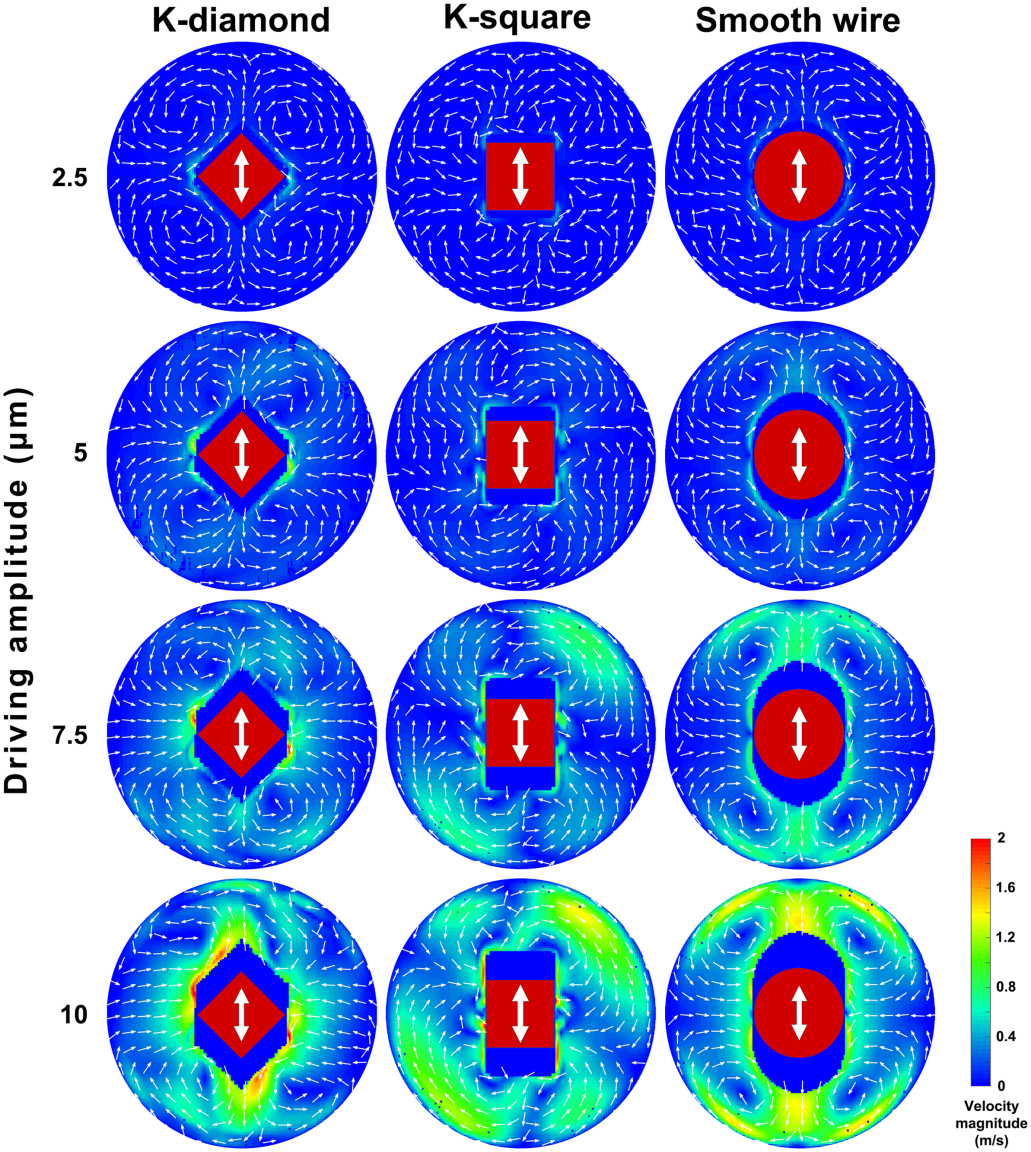
Time‐averaged irrigant velocity at a cross‐section of the root canal at the start of the twisted part of the K file (150 μm coronally to the tip) for different driving amplitudes (2.5, 5.0, 7.5 and 10 μm). The direction of main oscillation is depicted by the white arrows. The dark coloured area in the middle of each plot depicts the area occupied by the file/wire (in red) during oscillation. No time‐averaging took place in that area.

**FIGURE 8:**
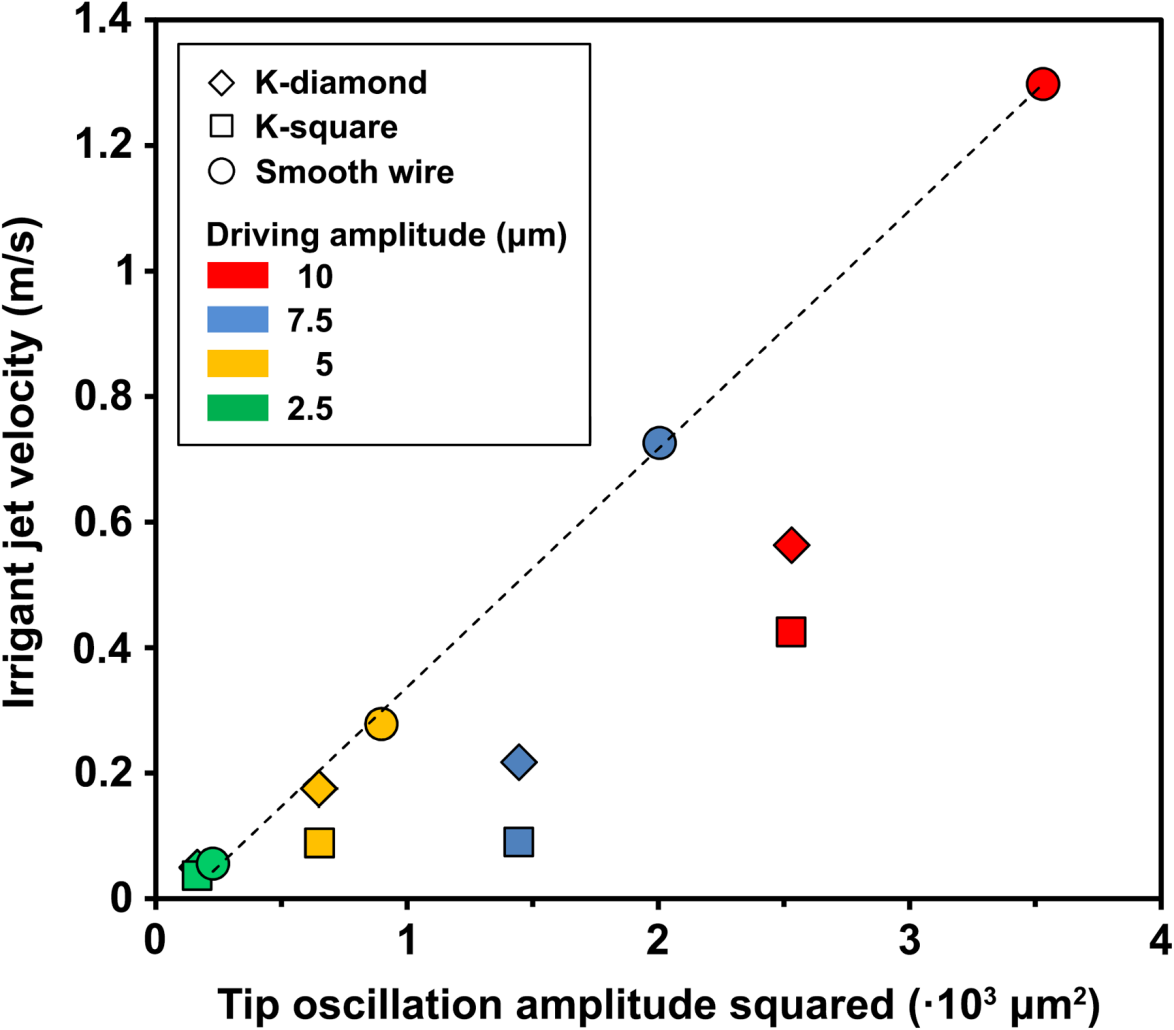
Time‐averaged irrigant jet velocity halfway between the root canal wall and the ultrasonic instrument along the direction of the main oscillation at a cross‐section of the root canal 150 μm coronally to the instrument tip (same as in Figure 7) as a function of the tip oscillation amplitude squared according to the numerical model. The irrigant jet velocity increases linearly with the square of the tip oscillation amplitude for the smooth wire (fitted dashed line, *R* ^2^ = 0.999), which scales with the analytical solution (Equation 1). The K‐files created a more complex flow and the highest jet velocities appeared at an angle to the direction of the main oscillation direction.

The ability of the numerical model to capture changes in the flow due to the increasing driving amplitude was assessed by comparison of the time‐averaged flow field at a cross‐section of the root canal 150 μm coronally to the file/wire tip (at the start of the twisted part of the K‐files). Differences were identified both between the various cases studied (K‐diamond, K‐square, smooth wire) and between the various driving amplitudes (Figure 7). The smooth‐wire cases showed a symmetric flow pattern that corresponded closely to the theoretical description of acoustic streaming around an ultrasonic file with a circular cross‐section (analytical solution) (Verhaagen et al., 2014). Steady jets of irrigant moving away from the file were noted along the direction of oscillation, while irrigant in‐flow took place along the direction perpendicular to the main oscillation direction. A quadrupolar flow with four steady vortices was formed around the file. The time‐averaged irrigant jet velocity increased linearly with the square of the tip amplitude (Figure 8), confirming the theoretical prediction of Equation 1. Steady jets, entrainment and vortices were also formed in the K‐diamond and K‐square cases but the flow was slightly asymmetric (Figure 7). The time‐averaged irrigant jet velocity also increased with the tip amplitude (Figure 8), although in the K‐square cases the highest jet velocity was found at 45° off the oscillation direction (along the diagonal of the square file cross‐section).

## DISCUSSION

The main purpose of this study was to develop and validate a three‐dimensional numerical model combining the oscillation of an ultrasonic file and the resulting irrigant flow along with their two‐way‐coupled interaction inside a root canal. While differences could also be observed, the predictions of the new model were in good agreement with the experiments conducted and the analytical solutions described previously (Verhaagen et al., 2014).

The numerical simulations showed that the viscous damping effect of the irrigant on the oscillation of a smooth wire was negligible. In addition, the flow pattern in this case was similar to the one calculated by an earlier two‐dimensional model that did not account for liquid viscous damping (Verhaagen et al., 2014). Therefore, one‐way FSI may be sufficient to describe the flow around an ultrasonic smooth wire. A direct comparison of our results to those reported by a previous study that also did not account for the damping effect of the irrigant (Chen et al., 2014) was not feasible because the geometry of the ultrasonic tip was different and details of its prescribed motion, and the resulting flow field were not reported in that study.

The damping effect became important when more vorticity was generated in the flow by the sharp twisted edges of the K‐files, which led to a 32% decrease in the tip oscillation amplitude when the irrigant and the root canal were taken into account. This difference emphasizes the importance of the two‐way‐coupled FSI used in the current model. The new 3D model also provides additional information about the flow in the apico‐coronal direction; the flow is inherently three‐dimensional, with irrigant flowing from the antinodes to the nodes along the file, thereby generating a preferred direction towards the apex due to the reduced distance between the file and the tapered root canal wall. Such details were missing from the earlier study (Verhaagen et al., 2014). Moreover, the twisting of the K‐file appeared to induce rotation of the flow around the file, causing the cross‐sectional flow field to be asymmetric (Figure 7). Despite the differences identified on individual cross‐sections of the root canal between K‐diamond and K‐square cases regarding the flow pattern, these cases gave very similar overall results, probably because ‘diamond’ and ‘square’ cross‐sections alternate every 0.256 mm along the K‐file (pitch = 2.045 mm). Therefore, tip orientation can be ignored in future experiments and numerical simulations, thereby reducing the number of cases that need to be examined.

Commercially available ultrasound devices include a feedback system which operates automatically for a brief period at the start‐up of each oscillation (~50 ms); this system takes into account the selected power setting, measures the load on the ultrasonic file and scans through a range of driving frequencies (approximately 28–32 kHz) in order to determine the optimum driving amplitude and frequency in each case (Verhaagen et al., 2012). The precise mapping of the feedback system is proprietary information beyond our control, so the driving frequency and amplitude were fixed in the numerical model. Nevertheless, its operation could explain a number of findings during the experiments, for instance, the increased oscillation amplitude of the K‐files inside the irrigant‐filled root canal compared with their free oscillation in air despite the viscous damping by the irrigant. This finding has also been described before (Verhaagen et al., 2012). Moreover, the driving frequency may have been different to some extent between the experiments and the simulations due to the action of the feedback system, leading to slight differences in the location and amplitude of the antinodes. The action of the feedback system may also be partially responsible for the transient effects (increased oscillation amplitude and more intense streaming) that have been observed during the start‐up phase (~50 ms) of ultrasonic activation (Jiang et al., 2010b; Malki et al., 2012). It has been reported that file oscillation stabilizes much faster (~3 ms) when driven at a constant amplitude and frequency without any feedback system (Verhaagen et al., 2012). Our numerical simulations, in which the files were driven at a constant amplitude and frequency, revealed that transient oscillation effects disappeared after the first two cycles (*t* = 0.066 ms) and that the flow pattern was stabilized by the 20th cycle (*t* = 0.656 ms). Therefore, the simulations were carried out for at least 20 cycles to ensure cost‐effective use of the computational resources.

In the process of model validation, it is necessary to consider all potential sources of error, both in the experiments and in the numerical simulations (Oberkampf & Trucano, 2002). Accurate vibrometer measurements near the tip of the K‐file are known to be challenging due to the size of the tip (Verhaagen et al., 2012). In addition, small deviations in the material properties that were used in the numerical model compared with the actual files, manufacturing defects and tolerances regarding their geometry may have also contributed to the differences in the oscillation pattern found between the structural model and the vibrometer measurements, especially near the tip of the file. Minor misalignment of the ultrasonic file inside the root canal during experiments may have also resulted in a slightly different oscillation pattern.

Considerable efforts were made to create the same conditions in the experiments and in the numerical model, in order to reduce the potential sources of error. However, some differences could not be avoided. Sodium hypochlorite was assumed to be the irrigant in all numerical simulations; however, distilled water or tap water was used as irrigant in the experiments in order to prevent corrosion of metal parts and any effect on the surface of the artificial root canals. Nevertheless, while the chemical activity of the irrigant is disregarded here, sodium hypochlorite is an aqueous solution with physical properties very similar to those of water (Guerisoli et al., 1998; Lide, 2005), and they should therefore exhibit the same physical flow characteristics. Furthermore, ultrasonic files were placed at 1 mm from the canal terminus in the PIV experiments and in the simulations but they were placed at 3 mm in the vibrometer experiments to improve the reflection of the laser beam off the file tip. A pilot simulation indicated that the effect of this difference in the insertion depth on the oscillation pattern was negligible.

The new model focused on file‐induced microstreaming and did not account for the possible formation and collapse of bubbles within the irrigant during ultrasonic activation (inertial cavitation). This would have dramatically increased the complexity of the model and required the simulation of a three‐phase system with stochastic non‐linear non‐localized phenomena. At the driving amplitudes used in this study, it is likely that only a very small amount of inertial cavitation would occur (Macedo et al., 2014), which was also corroborated by the lack of visible cavitation bubbles in the high‐speed imaging experiments conducted in this study.

Even though ultrasonic activation is often used to clean and disinfect areas beyond the main root canal (Robinson et al., 2018; Rödig et al., 2010a; 2010b; Thomas et al., 2014), a simple root canal geometry without any irregularities was used in the experiments and simulations described here. This choice facilitated the manufacturing of accurate artificial canals with sufficient transparency to allow both PIV and scanning laser vibrometry and also allowed a comparison of model predictions and experimental measurements to previously published analytical solutions that were possible to derive only for simple geometries (Verhaagen et al., 2014). As a next step, the model can be used to obtain information on the extent of microstreaming, the pressure and the shear stress applied to the root canal wall in more clinically relevant cases.

## CONCLUSIONS

A novel three‐dimensional numerical model combining the oscillation of an ultrasonic file and the created fluid flow along with their two‐way interactions inside a root canal was developed and validated by comparison to experiments and to theoretical solutions of the flow. This model can be used to predict the flow pattern under similar conditions and in similar flow domains.

## AUTHOR CONTRIBUTIONS

C. Boutsioukis, M. Versluis and L.W.M. van der Sluis conceptualized and designed the study. C. Boutsioukis and B. Verhaagen acquired the data. C. Boutsioukis, B. Verhaagen, M. Versluis, L.W.M. van der Sluis analysed and interpreted the data. C. Boutsioukis drafted the manuscript. C. Boutsioukis, B. Verhaagen, M. Versluis and L.W.M. van der Sluis critically revised the manuscript. C. Boutsioukis, L.W.M. van der Sluis and M. Versluis acquired the funding.

## CONFLICT OF INTEREST

The authors deny any conflicts of interest related to this study.

## Supporting information


Appendix S1

**Movie S1** Contours of instantaneous velocity magnitude near the tip of the K‐file along the main oscillation plane (*y*–*z* plane) during the 19th and 20th oscillation cycle according to numerical simulations (K‐diamond orientation, driving amplitude = 5 μm).

## Data Availability

The data that support the findings of this study are available from the corresponding author upon reasonable request.
