# Supplementary material for: Irrigant flow in the root canal during ultrasonic activation: A numerical fluid–structure interaction model and its validation
Source: Int Endod J. 2022 Jul 14;55(9):938–49. doi: 10.1111/iej.13791 (PMC9545492; doi:10.1111/iej.13791)
Supplement: Supplementary file 1 — Appendix S1 [file IEJ-55-938-s001.pdf]

SUPPLEMENTARY INFORMATION

Elliptical motion of the files

Despite the fact that all files/wires were driven along a single plane (*y*-direction) in the numerical model, a small lateral component (*x*-direction) was noted in the oscillation of the K-files leading to a slightly elliptical motion of their tip. This component appeared both when oscillating without any confinement (~2% of the oscillation in the *y*-direction) and in the confinement of the root canal (~3% of the oscillation in the *y*-direction) and it was possibly related to the twisting of the K-files (Figure S1). The lateral component was much smaller for the smooth wire (~0.5% of the oscillation in the *y*-direction). Sideways motion of ultrasonic files has also been reported before during experiments in a water tank (Verhaagen et al., 2012), though at larger amplitudes.

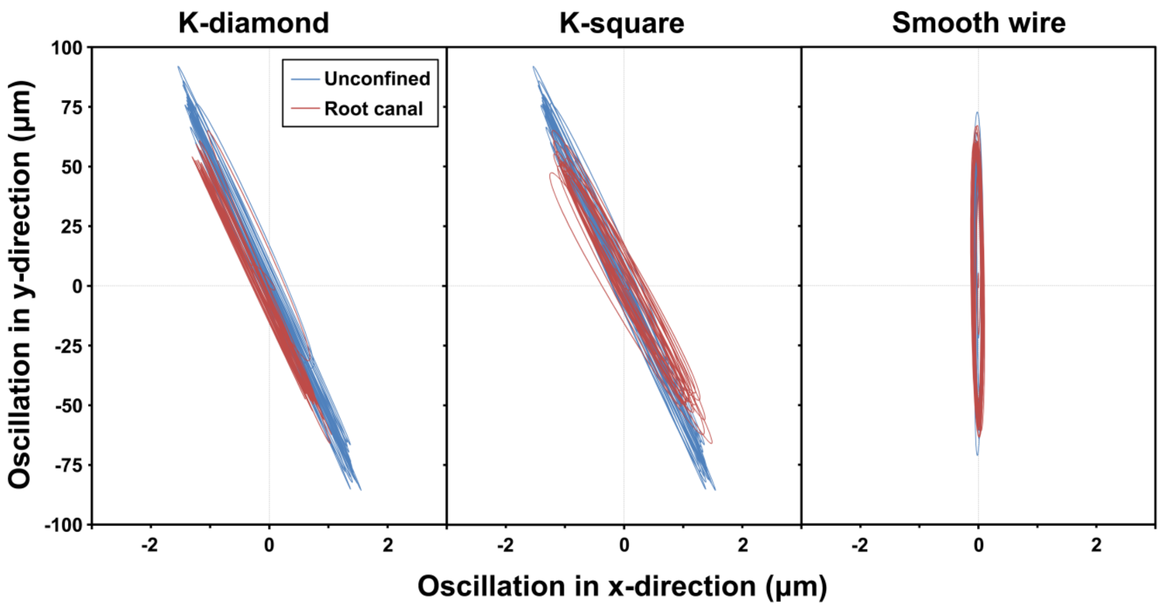

Figure S1: Tip oscillation pattern during the first 20 cycles when the files/wires oscillated unconfined or in the confinement of the root canal according to numerical simulations (driving amplitude = 10 µm). A different scale has been used in the horizontal and vertical axii to highlight the small lateral component (*x*-direction) in the oscillation of the K-files that led to a slightly elliptical motion.

Pressure and shear stress on the root canal wall

An examination of the time-averaged irrigant pressure on the root canal wall along the main oscillation plane of the K-files (*y-z* plane) according to numerical simulations revealed pressure peaks near the tip and the second antinode (Figure S2) that match the high time-averaged velocity areas observed in the same plane (Figure 6). The pressure peaks were mainly found near the tip in the smooth wire cases and also corresponded to high-velocity areas. Time-averaged shear stress peaks were also noted on the root canal wall near the tip and between the tip and the second antinode, for all three types of files/wires (Figure S2). These peaks were not in the region of the highest flow velocities, in agreement with the analytical solution showing that the maximum shear stress is found outside the center of the jet (Verhaagen et al., 2014). The magnitude of all pressure and shear stress peaks increased as a function of driving amplitude.

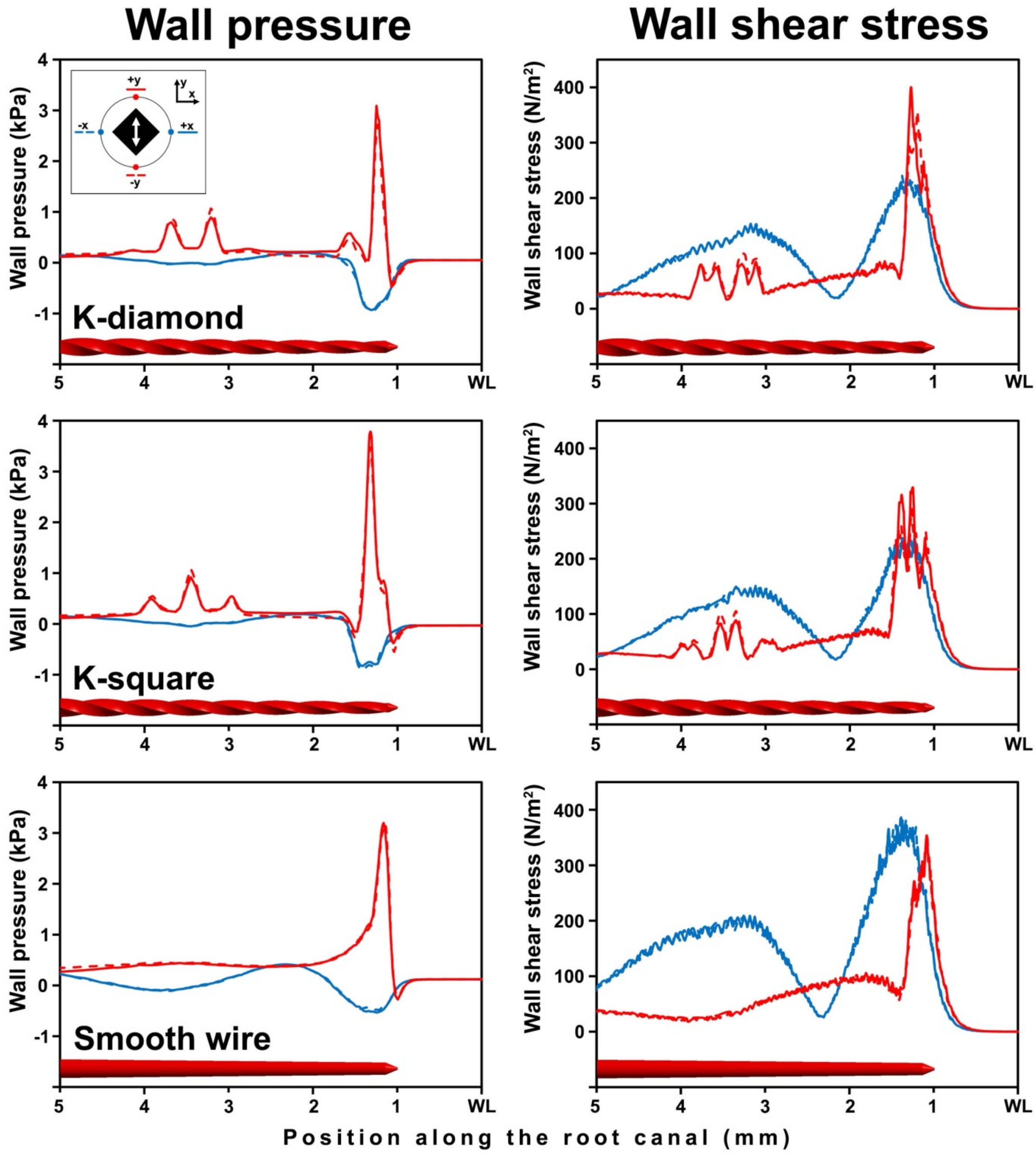

Figure S2: Time-averaged irrigant pressure and shear stress along the root canal wall at positions ±*y*, ±*x* (depicted in the inset) for the three different types of files/wires (driving amplitude = 10 µm). The inset shows a cross-section of the root canal and the ultrasonic file (not to scale). The white arrows indicate the main oscillation direction. The pressure peaks near the tip and, for the K-file, at 2-3 mm from the tip match the high velocity areas in the side view of Figure 6.
